# Pre-existing interstitial lung disease as a risk factor for pneumonitis associated with ramucirumab and paclitaxel in patients with gastric cancer: The impact of usual interstitial pneumonia

**DOI:** 10.1371/journal.pone.0198886

**Published:** 2018-06-07

**Authors:** Nobuyuki Koyama, Sou Katayanagi, Shigeyuki Kawachi

**Affiliations:** 1 Department of Clinical Oncology, Tokyo Medical University Hachioji Medical Center, Hachioji-shi, Tokyo, Japan; 2 Department of Digestive and Transplantation Surgery, Tokyo Medical University Hachioji Medical Center, Hachioji-shi, Tokyo, Japan; Auburn University College of Veterinary Medicine, UNITED STATES

## Abstract

**Objective:**

Combination treatment with ramucirumab and paclitaxel shows significant efficacy in patients with advanced gastric cancer as a second-line standard therapy. However, limited information is available about the development of pneumonitis associated with this treatment in clinical practice. This study aimed to characterize this form of pneumonitis and identify the risk factors for its onset.

**Methods:**

We retrospectively analyzed the medical records of 44 patients with gastric cancer who received combination treatment with ramucirumab and paclitaxel from 2016 to 2017. Then, the clinicopathological characteristics of patients who developed treatment-related pneumonitis were evaluated and further compared with those of patients who did not.

**Results:**

Six patients (13.6%) developed pneumonitis within five treatment cycles, and in five cases, remission was observed after cessation of combination treatment alone. The onset of pneumonitis was independently associated with pre-existing interstitial lung disease (ILD) (p = 0.025; odds ratio = 206.4). Patients with pneumonitis showed reduced time to treatment failure (median 56 vs. 138 days; p = 0.008), as compared with those without pneumonitis. Most patients with pre-existing ILD with a usual interstitial pneumonia (UIP) pattern developed pneumonitis.

**Conclusions:**

In clinical practice, pneumonitis associated with the combination treatment of ramucirumab and paclitaxel was generally mild, but common. Patients with gastric cancer with pre-existing ILD, particularly those presenting with a UIP pattern, undergoing this combination treatment, should be carefully monitored for the development of treatment-related pneumonitis.

## Introduction

Gastric cancer (GC) is the fifth most common malignancy and the third leading cause of cancer death worldwide, despite recent advances in multimodal therapy [1]. In pharmacotherapeutic strategies, combination chemotherapy with platinum plus fluoropyrimidine is the first-line standard therapy for advanced GC [2–4]. A second-line therapy, involving combination treatment with ramucirumab (RAM), an anti-vascular endothelial growth factor receptor-2 (VEGFR-2) monoclonal antibody, and solvent-paclitaxel (PTX), also improves overall survival (OS) [5], on the basis of the beneficial outcomes of a global phase III trial and is currently commonly employed as the second-line regimen for patients with GC [6].

In that trial, however, patients who received the combination treatment had higher incidence rates of many adverse events than those who received PTX monotherapy [6]. Particularly, proteinuria, hypertension, hemorrhagic events, and gastrointestinal perforation, which are considered RAM-related adverse events, were more frequently identified in patients who received the combination treatment. Conversely, the incidence of treatment-related pneumonitis, a life-threatening adverse event, was lower in patients who received the RAM and PTX combination treatment (1.5%) than in those who received PTX monotherapy (2.1%) in the trial [6]. Another phase III trial on GC reported that RAM-related pneumonitis occurred in 0.4% of patients who received second-line RAM monotherapy [7]. Furthermore, in a phase III trial for previously-treated patients with non-small cell lung cancer (NSCLC), the incidence of treatment-related pneumonitis was higher in patients who received combination treatment with RAM and docetaxel (2.1%) than in patients who received docetaxel monotherapy (1.6%) [8]. These findings suggest that the addition of RAM may increase the incidence of treatment-related pneumonitis. Considering these conflicting results from previous studies and the limited evidence for RAM-related pneumonitis, an optimal strategy for RAM-based treatment of GC needs to be established, which requires characterization of this form of pneumonitis and identification of the risk factors for its onset.

The incidence of treatment-related pneumonitis in some cancer types may be increased in patients with an underlying, pre-existing interstitial lung disease (ILD) [9–11]. ILDs comprise diverse types of diffuse parenchymal diseases with pathophysiological heterogeneity; among these, idiopathic interstitial pneumonias (IIP) of unknown etiology are further subdivided according to the American Thoracic Society (ATS)/European Respiratory Society (ERS)/Japanese Respiratory Society (JRS) statement [12]. Each IIP subtype presents different clinicopathological features, including imaging and pathological findings, clinical courses, and prognoses. Of the IIP subtypes, a usual interstitial pneumonia (UIP) pattern accounts for 80–90% of cases with IIP, and has a worse prognosis, with a median of 3–5 years’ survival, due to a lack of therapeutic options, drug resistance, and the frequency of acute exacerbation, than other types of IIP [12–17]. The IIP classification is also applicable to cases of treatment-related pneumonitis. Therefore, for the assessment of treatment-related pneumonitis, information about the presence or absence of pre-existing ILD, the subtype of ILD, and pneumonitis is needed.

In the present study, we retrospectively analyzed the medical records of patients with GC who received combination treatment with RAM and PTX and further evaluated the clinicopathological characteristics of those who developed treatment-related pneumonitis. To explore these characteristics particularly focusing on underlying pulmonary diseases including ILD and treatment-related pneumonitis may help to identify the risk factors for the onset of pneumonitis, and may facilitate prevention and early detection of such pneumonitis.

## Methods

### Patients

We retrospectively analyzed the medical records of 44 patients with GC who underwent combination treatment with RAM and PTX at Tokyo Medical University Hachioji Medical Center from 2016 to 2017. According to the treatment protocol in the pivotal phase III trial, the RAINBOW study, all patients received RAM (8 mg/kg) on days 1 and 15, and PTX (80 mg/m^2^) on days 1, 8, and 15, in a 28-day treatment regimen cycle [6]. Cessation or reduction of drug doses was determined by the attending physician, according to the onset of adverse events and disease progression. Chest X-ray examination was performed at day 1 of each treatment cycle, and chest CT scan was undertaken when a new shadow appeared. Chest and abdominal CT scans were performed each 2–3 months to evaluate the therapeutic effect of combination treatment with RAM and PTX. After data and information were blindly collected, patients were classified into those who did and those who did not develop pneumonitis during the course of combination therapy.

### Study assessment

After obtaining approval from our institutional review board (No. H–196), the patients’ medical records and computed tomography (CT) images were reviewed. The retrospective data were analyzed anonymously and patients were given the opportunity to opt out of this study. Thus, a waiver of written or oral informed consent was granted from the institutional review board. The maximal effect of the combination treatment was defined as complete response (CR), partial response (PR), stable disease (SD), or progressive disease (PD), according to the Response Evaluation Criteria in Solid Tumors (RECIST) guidelines [18]. The objective response rate (ORR) was defined as the proportion of patients who achieved CR or PR, and the disease control rate (DCR) was defined as the proportion of patients who achieved CR, PR, or SD. The therapeutic effect was evaluated on the basis of ORR, DCR, the time from the initiation of treatment with RAM and PTX to the confirmation of treatment failure (time to treatment failure; TTF), and death of the patient (OS). Adverse events associated with the combination therapy were confirmed by medical record review and were evaluated on the basis of the Common Terminology Criteria for Adverse Events (CTCAE) v4.0.

### Assessment of interstitial lung disease and drug-induced pneumonitis

Chest CT images of the study patients were reviewed. To assess treatment-related pneumonitis, we focused on newly developed shadows that presented diffuse parenchymal image patterns in patients undergoing RAM and PTX combination treatment. Treatment-related pneumonitis was diagnosed on the basis of negative results in sputum culture tests, serum β-D glucan levels, polymerase chain reaction assay for *Pneumocystis jirovecii* in sputum samples, and tests for viral antibodies. The image pattern of pre-existing ILD and treatment-related pneumonitis was categorized on the basis of the ATS/ERS/JRS statement [12].

### Statistical analysis

Chi-square and Mann–Whitney U tests were used to evaluate differences in clinicopathological characteristics between patient groups. Using the Kaplan–Meier method and the log-rank test, TTF and OS were calculated and compared between the groups. Potential confounding factors were assessed with the Cox proportional hazard model and multivariate logistic regression analysis. A *P* value < 0.05 was considered to indicate statistically significant differences.

## Results

### Patient characteristics

The incidence of treatment-related pneumonitis was 13.6%, which was more than eight-fold higher than that reported in a previous clinical trial (Table 1) [6]. Patients who developed treatment-related pneumonitis were all older than 70 years of age, and were older than those who did not develop pneumonitis. Patients with comorbid emphysema and ILD are classified into each category. Of pre-existing lung diseases, ILD occurred frequently in patients who developed treatment-related pneumonitis. The incidence of pneumonitis was significantly lower in patients who had emphysema, or no pre-existing lung diseases. Treatment courses showed no significant differences between patients who did or did not develop pneumonitis.

**Table 1:** Patient characteristics.

Characteristics		Total (n = 44)	Pneumonitis (+) (n = 6)	Pneumonitis (-) (n = 38)	p-value
Age (years-old)	Average ± SD	69.5 ± 11.0	76.3 ± 1.9	68.4 ± 11.5	0.020
	≥ 70	27 (61%)	6 (100%)	21 (55%)	
	< 70	17 (39%)	0 (0%)	17 (45%)	
Sex					0.365
	Male	34 (77%)	6 (100%)	28 (74%)	
	Female	10 (23%)	0 (0%)	10 (26%)	
Smoking history (pack-year)					0.598
	Never	16 (36%)	2 (33%)	14 (37%)	
	20 ≥	3 (7%)	1 (17%)	2 (5%)	
	< 20	25 (57%)	3 (50%)	22 (58%)	
Primary site					0.956
	Cardia	8 (18%)	1 (17%)	7 (18%)	
	Corpus	13 (30%)	2 (33%)	11 (29%)	
	Angle	10 (23%)	1 (17%)	9 (24%)	
	Antrum	11 (25%)	2 (33%)	9 (24%)	
	Pylorus	2 (5%)	0 (0%)	2 (5%)	
Histological type					0.742
	Papillary ad (pap)	2 (5%)	0 (0%)	2 (5%)	
	Tubular ad (tub)	16 (36%)	2 (33%)	14 (37%)	
	Poorly differentiated ad (por)	20 (45%)	4 (67%)	16 (42%)	
	Signet-ring cell carcinoma (sig)	5 (11%)	0 (0%)	5 (13%)	
	Mucinous ad (muc)	1 (2%)	0 (0%)	1 (3%)	
Surgical resection					0.676
	+	26 (59%)	3 (50%)	23 (61%)	
	-	18 (41%)	3 (50%)	15 (39%)	
Treatment line	Median		2	2	0.381
	2nd	28 (64%)	5 (83%)	23 (61%)	
	3rd	7 (16%)	0 (0%)	7 (18%)	
	≥ 4th	9 (20%)	1 (17%)	8 (21%)	
Treatment course	Median	4 (10%)	4	4	0.447
	≥ 4	19 (43%)	2 (33%)	17 (45%)	
	< 4	25 (57%)	4 (67%)	21(55%)	
Pre-existing pulmonary disease					0.039
	-	28 (64%)	2 (33%)	26 (68%)	
	ILD	6 (14%)	4 (67%)	2 (5%)	
	Emphysema	11 (25%)	1 (17%)	10 (26%)	
	Bronchiectasis	2 (5%)	0 (0%)	2 (5%)	
Pre-existing ILD					0.002
	+	6 (14%)	4 (67%)	2 (5%)	
	-	38 (86%)	2 (33%)	36 (95%)	
Subsequent treatment					0.664
	+	22 (50%)	4 (67%)	18 (47%)	
	-	22 (50%)	2 (33%)	20 (53%)	

SD, standard deviation; ad, adenocarcinoma; ILD, interstitial lung disease

### Outcomes of treatment with ramucirumab and paclitaxel

The ORR and DCR of RAM and PTX combination treatment were 20.5% and 65.9%, respectively, which were lower than the values reported in a previous phase III trial (27.9% and 80.0%, respectively) (S1 Table) [6]. Although patients who developed pneumonitis had a lower ORR (16.7% vs. 21.1%) and higher DCR (83.3% vs. 63.2%) than those who did not develop pneumonitis, there were no significant differences in the treatment efficacy between these patient groups, indicating no evidence of an association between pneumonitis and efficacy.

**Fig 1:**
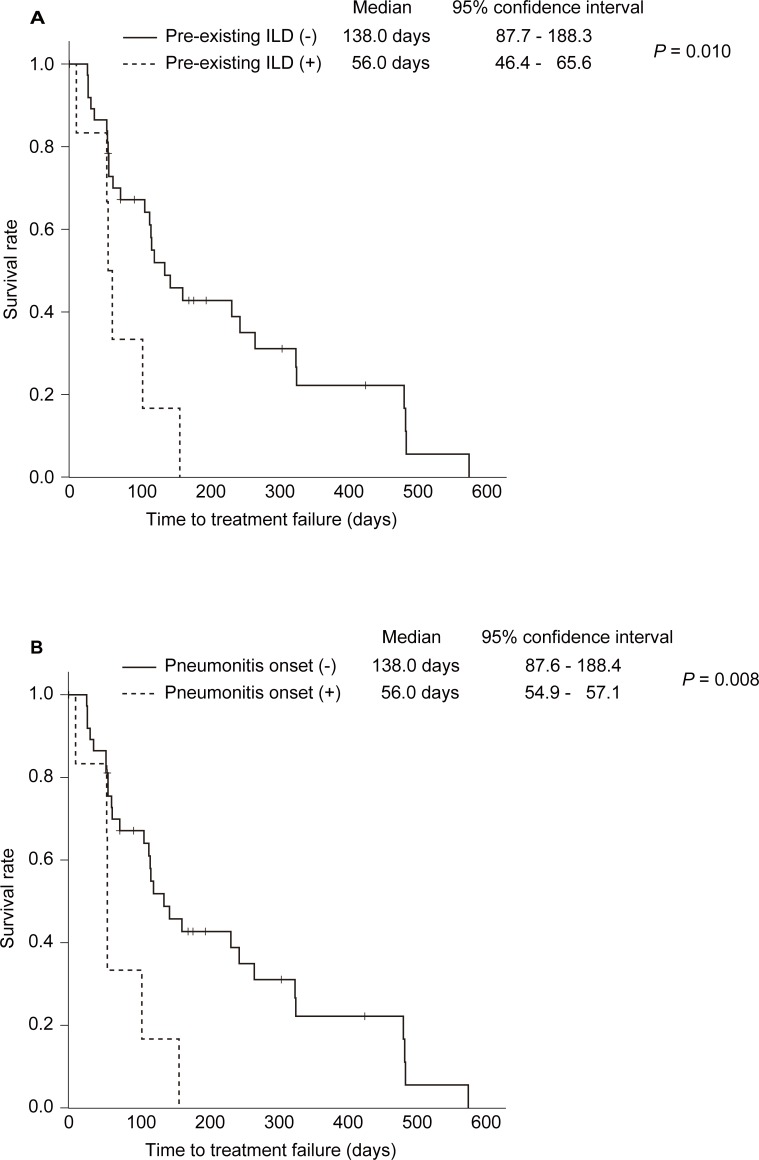
**Kaplan−Meier survival curves and log-rank tests of time to treatment failure in patients with gastric cancer, with and without pre-existing interstitial lung diseases (A) and patients with gastric cancer who did and did not develop pneumonitis associated with combination treatment with ramucirumab and paclitaxel (B)**.

The Kaplan–Meier survival curves and log-rank tests showed that patients with pre-existing ILD (p = 0.010; median 138 vs. 56 days) and those who developed pneumonitis associated with RAM and PTX combination treatment (p = 0.008; median 138 vs. 56 days) had significantly shorter TTF (Fig 1A and 1B). In a Cox proportional hazard model (Table 2), the onset of pneumonitis (p = 0.007; hazard ratio = 5.385) and treatment course (≥ four courses) (p < 0.001; hazard ratio = 0.040) were independently associated with TTF. The treatment course (p = 0.002; hazard ratio = 0.109) alone was independently associated with OS.

**Table 2:** Cox proportional hazard model.

Characteristics		p-value	Hazard ratio	95% Confidence interval
Time to treatment failure				
	Age (≥ 70 years-old vs. < 70 years-old)	0.428	0.688	0.273–1.735
	Smoking history (+ or -)	0.055	0.397	0.155–1.020
	Pre-existing ILD (+ or -)	0.115	2.888	0.773–10.799
	Pneumonitis onset (+ or -)	0.007	5.385	1.590–18.244
	Objective response (CR+PR)	0.292	0.510	0.146–1.787
	Treatment line (2nd line vs. other lines)	0.170	1.769	0.783–3.994
	Treatment course (≥ 4 courses vs. < 4 courses)	0.001 >	0.040	0.011–0.143
Overall survival				
	Age (≥ 70 years-old vs. < 70 years-old)	0.200	0.461	0.141–1506
	Smoking history (+ or -)	0.053	0.349	0.120–1.014
	Pre-existing ILD (+ or -)	0.108	3.256	0.773–13.720
	Pneumonitis onset (+ or -)	0.846	1.152	0.275–4.833
	Objective response (CR+PR)	0.835	0.861	0.212–3.506
	Treatment line (2nd line vs. other lines)	0.095	2.313	0.863–6.194
	Treatment course (≥ 4 courses vs. < 4 courses)	0.002	0.109	0.026–0.456

ILD, interstitial lung disease; CR, complete response; PR, partial response

### Adverse events associated with ramucirumab and paclitaxel

The profile of adverse events encountered in this study was similar to that in the previous phase III trial including hypertension, proteinuria, bleeding events, peripheral sensory neuropathy, constipation, pedal edema, and hematological adverse events (S2 Table) [6]. Pneumonitis was a common identifiable event. There were no significant differences in the incidence of adverse events between the two patient groups.

### Characteristics of pre-existing interstitial lung disease and treatment-related pneumonitis

In a logistic regression analysis aimed at identifying clinical factors that are independently associated with RAM and PTX combination treatment-related pneumonitis, only pre-existing ILD (p = 0.025; odds ratio = 206.431) was identified as a statistically significant factor (Table 3).

**Table 3:** Logistic regression analysis for pneumonitis associated with ramucirumab and paclitaxel combination treatment.

Characteristics	p-value	Odds ratio	95% Confidence interval
Pre-existing ILD	0.025	206.431	1.953–21819.073
Therapeutic response	0.107	0.101	0.006–1.642
Age	0.630	1.047	0.869–1.261
Smoking history	0.257	0.998	0.994–1.002
Treatment line	0.761	1.174	0.419–3.285
Treatment course	0.359	0.737	0.384–1.415

ILD, interstitial lung disease

Of six patients with pre-existing ILD, four patients presented with a UIP pattern (Table 4). Furthermore, three of the four patients developed pneumonitis associated with RAM and PTX. Most cases of pneumonitis were mild; four of six cases were grade 1 and regressed after cessation of RAM and PTX treatment alone. One patient developed grade 3 pneumonitis, which showed a diffuse alveolar damage pattern. Median time from the initiation of combination treatment with RAM and PTX to the onset of pneumonitis was 56 days (range, 10 to 160 days).

**Table 4:** Characteristics of pre-existing interstitial lung disease and pneumonitis associated with ramucirumab and paclitaxel combination treatment.

	Image pattern		CTCAE Grade	Time to onset (days)	Treatment
	Pre-existing ILD	Pneumonitis			
Patient 1	cNSIP				
Patient 2	RB-ILD	COP	1	160	Only cessation of RAM and PTX
Patient 3	UIP				
Patient 4	UIP	cNSIP	1	106	Only cessation of RAM and PTX
Patient 5	UIP	DAD	3	10	Cessation of RAM and PTX, oxygen inhalation, and corticosteroid therapy
Patient 6	UIP	cNSIP	2	56	Cessation of RAM and PTX and oxygen inhalation
Patient 7		COP	1	56	Only cessation of RAM and PTX
Patient 8		HP like	1	55	Only cessation of RAM and PTX

ILD, interstitial lung disease; CTCAE, common terminology criteria for adverse events v4.0; cNSIP, cellular non-specific interstitial pneumonia; RB-ILD, respiratory bronchiolitis-associated interstitial lung disease; COP, cryptogenic organizing pneumonia; RAM, ramucirumab; PTX, paclitaxel; UIP, usual interstitial pneumonia; DAD, diffuse alveolar damage; HP, hypersensitivity pneumonitis

## Discussion

In the present study, pneumonitis associated with RAM and PTX combination treatment occurred more commonly than reported in a previous clinical trial [6]. In these patients, the onset of pneumonitis was independently associated with pre-existing ILD. Patients with pre-existing ILD were older than those without pre-existing ILD in univariate analysis (data not shown). Although patients who developed pneumonitis were also older than those who did not in univariate analysis, multivariate logistic regression analysis showed no association between age and the onset of pneumonitis. Although no information on pre-existing ILD was provided in that trial report [6], a previous retrospective study of patients with NSCLC with pre-existing ILD showed a high incidence of pneumonitis associated with PTX monotherapy (21.4%) [19]. These results indicate that pre-existing ILD is potently associated with the onset of pneumonitis. On the other hand, in this study, Kaplan–Meier survival curves and log-rank tests showed that pre-existing ILD is also associated with TTF and OS, along with the onset of pneumonitis. However, a Cox proportional hazard model showed no independent associations between pre-existing ILD and TTF or OS. Thus, both pre-existing ILD and the onset of pneumonitis are significantly associated with TTF and OS individually, whereas the onset of pneumonitis may be more directly associated with TTF and OS than pre-existing ILD. The findings of the present study suggest that pre-existing ILD may be a risk factor for the onset of pneumonitis associated with RAM and PTX combination treatment in patients with GC. Furthermore, pre-existing ILD and the onset of pneumonitis may be predictive factor for TTF and OS individually. In particular, the onset of pneumonitis was independently associated with TTF, but not with OS. Most pneumonitis events were mild and in fact, more than half of patients who developed pneumonitis underwent subsequent chemotherapies; hence, the onset of pneumonitis may cause the cessation of the combination treatment but may not be an independent prognostic factor for OS.

Pneumonitis associated with combination treatment with RAM and PTX was predominantly found in patients with pre-existing ILD with a UIP pattern according to the IIP classification, even though a limited number of patients and no statistical significance. Patient with IIP with a UIP pattern has a worse prognosis compare with those with other types of IIP [12–17]. Kenmotsu et al. reported that patients with lung cancer and ILD with a UIP pattern showed a higher incidence of chemotherapy-related exacerbation of ILD than that of patients with ILD with a non-UIP pattern [20]. However, to the best of our knowledge, association between treatment-related pneumonitis in patients with GC and the UIP subtype of pre-existing ILD has not been reported to date. A predominant UIP pattern in pre-existing ILD may have an impact on the onset of cancer treatment-related pneumonitis, regardless of the type of cancer.

The present study had a few limitations. First, this was a small retrospective study; hence, it is potentially subject to bias. Although multivariate statistical analyses were conducted to adjust potential confounding factors, independent associations of treatment courses with TTF and OS may be confounded by reverse causality. Second, in terms of the drug causing the treatment-related pneumonitis, it remains unclear whether RAM, PTX, or both drugs are responsible for the onset of pneumonitis. In a previous phase III trial, pneumonitis associated with RAM monotherapy was identified in only one patient [7]. RAM and PTX combination therapy is commonly employed in clinical practice, and all patients in this study received RAM and PTX combination treatment in this study. Thus, pneumonitis associated with the combination therapy, rather than with each monotherapy, may need to be characterized. Usui et al. demonstrated a higher incidence of pneumonitis in patients with colorectal cancer who were treated with conventional cytotoxic chemotherapeutic drugs plus bevacizumab (BEV), another VEGF inhibitor, than in those who did not receive BEV [21]. Furthermore, Sekimoto et al. reported a patient with NSCLC who was treated with a combination of carboplatin, PTX, and BEV [22]. The combination of a VEGF inhibitor with a cytotoxic anti-tumor drug may have an impact on the onset of treatment-related pneumonitis. A large-scale prospective study of treatment with RAM and PTX in patients with GC with pre-existing ILD is therefore warranted.

## Conclusions

The present study identified the presence of pre-existing ILD as a risk factor for pneumonitis associated with RAM and PTX combination treatment, a second-line standard pharmacotherapy in patients with GC. Furthermore, a UIP pattern of pre-existing ILD may have an impact on the onset of this treatment-related pneumonitis. Patients with GC with pre-existing ILD, particularly those presenting with a UIP pattern, undergoing combination treatment with RAM and PTX, should be carefully monitored for the development of treatment-related pneumonitis.

## Supporting information

S1 Table Therapeutic response to ramucirumab and paclitaxel combination treatment. (DOCX)

S2 Table Adverse events associated with ramucirumab and paclitaxel combination treatment. (DOCX)
