# Supplementary material for: Pre-existing interstitial lung disease as a risk factor for pneumonitis associated with ramucirumab and paclitaxel in patients with gastric cancer: The impact of usual interstitial pneumonia
Source: PLoS One. 2018 Jun 7;13(6):e0198886. doi: 10.1371/journal.pone.0198886 (PMC5991747; doi:10.1371/journal.pone.0198886)
Supplement: S1 Table — (DOCX) [file pone.0198886.s001.docx]

| **S1 Table. Therapeutic response to ramucirumab and paclitaxel combination treatment.** | | | | | |
| --- | --- | --- | --- | --- | --- |
| **Therapeutic response** | | **Total (n = 44)** | **Pneumonitis (+) (n = 6)** | **Pneumonitis (-) (n = 38)** | **p-value** |
|  | **Complete response** | 0 | 0 | 0 | 0.580 |
|  | **Partial response** | 9 | 1 | 8 |  |
|  | **Stable disease** | 20 | 4 | 16 |  |
|  | **Progressive disease** | 15 | 1 | 14 |  |
|  | **Objective response rate** | 20.5% | 16.7% | 21.1% | 0.805 |
|  | **Disease control rate** | 65.9% | 83.3% | 63.2% | 0.333 |
