# Supplementary material for: Pre-existing interstitial lung disease as a risk factor for pneumonitis associated with ramucirumab and paclitaxel in patients with gastric cancer: The impact of usual interstitial pneumonia
Source: PLoS One. 2018 Jun 7;13(6):e0198886. doi: 10.1371/journal.pone.0198886 (PMC5991747; doi:10.1371/journal.pone.0198886)
Supplement: S2 Table — (DOCX) [file pone.0198886.s002.docx]

| **S2 Table Adverse events associated with ramucirumab and paclitaxel combination treatment.** | | | | | |
| --- | --- | --- | --- | --- | --- |
| **Adverse events** | | **Total (n = 44) (%)** | **Pneumonitis (+) (n = 6) (%)** | **Pneumonitis (-) (n = 38) (%)** | **p-value** |
| **Non hematological AE (Grade ≥ 2)** | |  |  |  |  |
|  | **Hypertension** | 10 (22.7%) | 2 (33.3%) | 8 (21.1%) | 0.606 |
|  | **Proteinuria** | 12 (27.3%) | 2 (33.3%) | 10 (26.3%) | 0.658 |
|  | **Nasal bleeding** | 2 (4.5%) | 0 | 2 (5.3%) | 1.000 |
|  | **Upper GI bleeding** | 1 (2.3%) | 0 | 1 (2.6%) | 1.000 |
|  | **Hepatotoxicity** | 3 (6.8%) | 0 | 3 (7.9%) | 1.000 |
|  | **Appetite loss** | 8 (18.2%) | 0 | 8 (21.1%) | 0.573 |
|  | **Skin rash** | 1 (2.3%) | 0 | 1 (2.6%) | 1.000 |
|  | **Peripheral sensory neuropathy** | 5 (11.4%) | 1 (16.7%) | 4 (10.5%) | 0.538 |
|  | **Fatigue** | 3 (6.8%) | 0 | 3 (7.9%) | 1.000 |
|  | **Alopecia** | 2 (4.5%) | 0 | 2 (5.3%) | 1.000 |
|  | **Colonic obstruction** | 2 (4.5%) | 0 | 2 (5.3%) | 1.000 |
|  | **Stomatitis** | 12 (27.3%) | 2 (33.3%) | 10 (26.3%) | 0.663 |
|  | **Constipation** | 6 (13.6%) | 2 (33.3%) | 4 (10.5%) | 0.182 |
|  | **Pedal edema** | 7 (15.9%) | 1 (16.7%%) | 6 (15.8%) | 1.000 |
|  | **Pigmentation** | 2 (4.5%) | 1 (16.7) | 1 (2.6%) | 0.257 |
|  | **Fever** | 1 (2.3%) | 0 | 1 (2.6%) | 1.000 |
|  | **Pruritus** | 1 (2.3%) | 0 | 1 (2.6%) | 1.000 |
|  | **Emboli of splenic vein** | 2 (4.5%) | 0 | 2 (5.3%) | 1.000 |
|  | **Gastric perforation** | 4 (9.1%) | 0 | 4 (10.5%) | 1.000 |
| **Hematological AE (Grade ≥ 3)** | |  |  |  |  |
|  | **Neutropenia** | 17 (38.6%) | 3 (50.0%) | 14 (36.8%) | 0.662 |
|  | **Anemia** | 4 (9.1%) | 1 (16.7%) | 3 (7.9%) | 0.456 |
|  | Thrombocytopenia | 3 (6.8%) | 1 (16.7%) | 2 (5.3%) | 0.363 |
| AE, adverse event | |  |  |  |  |
